# Atherosclerotic plaque in carotid arteries in systemic lupus erythematosus: frequency and associated risk factors

**DOI:** 10.1590/S1516-31802005000300010

**Published:** 2005-05-02

**Authors:** Alexandre Wagner Silva de Souza, Francisca Satomi Hatta, Fausto Miranda, Emília Inoue Sato

**Keywords:** Systemic lupus erythematosus, Cardiovascular diseases, Ultrasonics, Carotid artery diseases, Arteriosclerosis, Lúpus eritematoso sistêmico, Doenças cardiovasculares, Ultra-som, Doenças das artérias carótidas, Arteriosclerose

## Abstract

**CONTEXT AND OBJECTIVE:**

Atherosclerotic disease is an important cause of morbidity and mortality in systemic lupus erythematosus (SLE) patients. No previous study has estimated carotid disease prevalence in such patients in Brazil. The aim was to evaluate the prevalence of atherosclerotic plaque in carotid arteries, in SLE patients and controls, and to verify possible associations between risk factors and carotid plaque.

**DESIGN AND SETTING:**

Cross-sectional study, at Universidade Federal de São Paulo — Escola Paulista de Medicina.

**METHODS:**

Carotid plaque prevalence was assessed by B-mode ultrasound in 82 female SLE patients of mean age 34.0 years and 62 controls of mean age 35.7 years. Plaque was defined as a distinct area of hyperechogenicity and/or focal protrusion of the vessel wall into the lumen. Risk factors for coronary disease and SLE-related variables were determined.

**RESULTS:**

50% of patients and 29% of controls presented carotid plaque. Older age, longer disease duration, higher Systemic Lupus International Collaborating Clinics (SLICC) score, higher levels of low-density lipoprotein and greater diabetes, obesity, premature ovarian failure and family history of coronary artery disease were found in patients with carotid plaque than in those without plaque. Patients with plaque were younger than controls with plaque. SLE diagnosis, obesity, older age, higher SLICC score and longer disease duration were independent risk factors for carotid plaque.

**CONCLUSION:**

Young patients with SLE present higher prevalence of carotid plaque than controls. SLE diagnosis was a significant risk factor for carotid atherosclerosis.

## RESUMO

**CONTEXTO E OBJETIVO:**

Doença aterosclerótica é importante causa de morbidade e mortalidade em pacientes com lúpus eritematoso sistêmico. Não há estudos que verifiquem a prevalência de aterosclerose em carótidas nesses pacientes no Brasil. O objetivo deste trabalho foi estimar a prevalência de aterosclerose em carótidas em pacientes com lúpus e em controles, e avaliar associação entre fatores de risco e aterosclerose em carótidas.

**TIPO DE ESTUDO E LOCAL:**

Estudo transversal na: Universidade Federal de São Paulo - Escola Paulista de Medicina, São Paulo, Brasil.

**MÉTODOS:**

Foi utilizado o ultra-som modo B de carótidas em 82 mulheres com lúpus eritematoso sistémico e em 62 controles. A idade média das pacientes foi de 34,0 anos (variação 16-65) e dos controles foi de 35,7 anos (variação 18-64). Placa aterosclerótica foi definida como área distinta de hiperecogenicidade ou como área focal de protrusão da parede para o lúmen do vaso. Foram pesquisados fatores de risco para doença coronária e variáveis relacionadas ao lúpus.

**RESULTADOS:**

50% dos pacientes e 29% dos controles apresentaram placas em carótidas (p = 0,011). Idade mais avançada, maior tempo de lúpus, maior pontuação do escore Systemic Lupus International Collaborating Clinics, maiores níveis de lipoproteína de baixa densidade, maior freqüência de diabetes, falência ovariana precoce, obesidade e história familiar de doença coronariana foram encontrados em pacientes com placas de carótidas. Pacientes com placas em carótidas tiveram menor média de idade em relação às controles com placas. Fatores de risco associados a placas em carótidas foram diagnóstico de lúpus (razão de chances: 4,74; intervalo de confiança de 95%: 1,83 a 12,27), obesidade (razão de chances: 6,16; intervalo de confiança de 95%: 1,84 a 20,64), idade avançada (razão de chances: 1,12; intervalo de confiança de 95%: 1,07 a 1,17), maior escore Systemic Lupus International Collaborating Clinics (razão de chances: 1,69; intervalo de confiança de 95%: 1,31 a 2,52) e maior tempo de lúpus (razão de chances: 1,65; intervalo de confiança de 95%: 1,06 a 2,56).

**CONCLUSÃO:**

Pacientes jovens com lúpus apresentam maior prevalência de aterosclerose de carótidas. O diagnóstico de lúpus eritematoso sistêmico foi fator de risco para aterosclerose de carótidas.

## INTRODUCTION

Over recent decades, the survival rates for patients with systemic lupus erythematosus have improved considerably, and the five-year survival reached 80-90% in the 1990s.^1^ Accelerated atherosclerosis is one of the emerging problems facing such patients, following this improvement in their survival. The prevalence of angina pectoris and acute myocardial infarction in different cohort studies on lupus patients has been found to range from 6.6% to 10% and mainly affect younger women with a mean age of less than 50 years.^2-4^ In Manzi's study, women with systemic lupus aged between 35 and 44 years presented a 52fold greater risk of developing myocardial infarction than did women of similar age without this disease, in the Framingham Offspring Study.^4^

Clinical events are only part of the atherosclerotic burden. The inclusion of subclinical disease in the assessment of atherosclerosis seems to reflect the true extent of the problem.^5^ In the general population, the detection of subclinical disease has been found to be an independent predictive factor for future atherosclerotic events.^6^ High-resolution B-mode carotid ultrasound has been shown to be an important tool for epidemiological research in a general population and in different high-risk groups.^7-10^ The presence of carotid plaques is indicative of generalized atherosclerosis.^11^

There are few studies that have evaluated carotid disease in women with systemic lupus erythematosus and they have shown the presence of atherosclerotic plaque in 32 to 41% of the patients. All these studies were performed in developed countries with large predominance of Caucasian racial type.^12-16^

The objective of the present study was to evaluate the prevalence of carotid plaque in female patients with systemic lupus erythematosus and in controls. The secondary objective was to analyze the potential association between carotid plaque and traditional risk factors for coronary artery disease, and also between carotid plaque and risk factors related to lupus, such as disease duration, Systemic Lupus International Collaborating Clinics damage index,^17^ anticardiolipin antibodies and the use of prednisone and antimalarial drugs, in a multiracial population from a developing country.

## METHODS

### Patients and controls

Eighty-two consecutive patients who fulfilled the updated American College of Rheumatology criteria for systemic lupus erythematosus^18^ and were regularly being followed up at the Rheumatology Division Outpatient Clinic at the Universidade Federal de São Paulo were randomly invited to participate in the study when they came to the medical appointment in the outpatient clinic. The selection period began in September 2000 and ended in November 2001 and, during this period, all patients who were on regular follow-up in the outpatient clinic were invited. No restrictions concerning age or risk factors were applied to the selection process.

The control group consisted of sixty-two women whose ages were similar to the patients' ages. They were randomly invited from homes and workplaces near to the Universidade Federal de São Paulo and the university's employees. Controls who had previously had an autoimmune disease were excluded. The local Ethics Committee approved the study's design and all participants gave their written informed consent before being enrolled in the study.

### Measurements of variables

All participants underwent an interview, a physical examination and a carotid ultrasound scan. Blood samples were collected from all participants after 12 hours of fasting. Plasma glucose, total cholesterol, high-density lipoprotein cholesterol (HDLc), low-density lipoprotein cholesterol (LDL-c), and triglycerides determinations were performed by enzymatic assay. The Friedwald equation was used to estimate LDL-c. Anticardiolipin antibodies were determined by enzyme-linked assay and results were considered positive when greater than 10 GPL units or 10 MPL units.

The traditional risk factors for coronary artery disease that were considered were: age over 55 years for women, current smoking, high blood pressure (above 140 × 90 mmHg or use of any antihypertensive drug), family history of premature coronary disease in a first-degree relative before the age of 65 for women and 55 for men, diabetes mellitus, high density lipoprotein lower than 40 mg/dl, low density lipoprotein higher than 160 mg/dl and any previous atherosclerotic disease (stroke, myocardial infarction, angina pectoris, myocardial bypass or angioplasty, peripheral arteriopathy or aorta aneurysm), in accordance with the National Cholesterol Education Program (Panel III).^19^ Obesity (body mass index greater than 30 kg/m^2^) was also considered to be a risk factor for coronary disease in this study.^20^ Premature ovarian failure was considered to be a risk factor when the last natural menstruation occurred before the age of 45 years.^21^

The interview and the patient's clinical records were used to collect information about the duration of systemic lupus erythematosus, the menopausal age, duration and cumulative prednisone dose and duration of antimalarial drug use. The Systemic Lupus International Collaborating Clinics index was applied. This is widely used in studies for evaluating cumulative damage to organs and systems in patients with systemic lupus erythematosus.^17^

### Carotid ultrasound measurements

Ultrasound scans were performed at the Flow Laboratory of the Vascular Division of the Universidade Federal de São Paulo. Highresolution B-mode ultrasound equipment (Advanced Technology Laboratories Ultramark-9) with a 7-MHz linear-array imaging probe was used. A trained blinded sonography operator performed all the scans.

The scans included the right and left common carotid arteries, carotid bulb and the first 1.5 cm of the internal and external carotid arteries. The vessels were imaged using multiple planes, and the observer searched for focal plaques. Plaque was defined either as a distinct area of hyperechogenicity and/or a focal protrusion of the vessel wall into the lumen. All scans were recorded onto JVC high-resolution Super VHS-120 tapes, using a JVC HR-596004 high-resolution Super VHS video cassette recorder, and were analyzed later.

### Statistical methods

Two-sample Student's t tests were used to analyze differences between patients and controls for normally distributed variables. The chi-squared and Fisher exact tests were used to evaluate differences between category data for patients and controls. The Pearson correlation coefficient was used for associations between the number of risk factors for coronary artery disease and age, in both groups, and between the number of those risk factors and disease duration in patients with lupus. Fifty-three scans were randomly re-analyzed by the same observer to assess the reproducibility of the results and, from this, the K statistics reflected substantial agreement (0.64).

Two multiple forward logistic regression models were built to analyze associations between carotid focal plaque and different risk factors for coronary artery disease. In the first model, patients and controls were included. The independent variables were age at the time of the study, presence of diabetes, presence of hypertension, low level of high-density lipoprotein, high level of low-density lipoprotein, premature family coronary disease, tobacco use, history of previous atherosclerotic events, obesity and the diagnosis of systemic lupus erythematosus. Variables related to systemic lupus were used in the second logistic regression model for associations with carotid plaque. The variables included were disease duration in years, the Systemic Lupus International Collaborating Clinics damage score, duration of antimalarial drug and prednisone use, and cumulative prednisone dose.

Values of p < 0.05 were considered significant.

## Results

The mean age of the patients was 34.0 (± 11.7), ranging from 16 to 65 years, and for the controls it was 35.7 years (± 10.9), ranging from 18 to 64 years (p = 0.382). Only 2 patients (2.4%) and 3 controls (4.8%) were older than 55 years (p = 0.652). The duration of lupus ranged from 3 to 276 months and the median was 60 months. Fifty percent of the patients and 56.5% of the controls were white (p = 0.663). The mean Systemic Lupus International Collaborating Clinics score was 1.6 (± 1.5), ranging from 0 to 6.

The most common risk factors among the patients were hypertension (37.8%), high level of low-density lipoprotein (20.7%) and obesity (19.5%). In the control group, family history of premature coronary artery disease (30.6%), high level of LDL-c (25.8%) and low level of HDL-c (25.8%) were most common (Table 1). Premature ovarian failure was found in nine patients (10.9%) and in four controls (6.4%) (p = 0.348). The mean number of risk factors for coronary disease was 1.97 (± 1.78) in the patients and 1.61 (± 1.32) in the control group (p = 0.163). At least one traditional risk factor for coronary disease was found in 75.6% of the patients and in 77.4% of the controls (p = 0.628). Twenty-three percent of the patients and 24% of the controls had only one risk factor (p = 0.980), and 37.8% of the patients and only 17.7% of the controls had 3 or more risk factors (p = 0.005). Previous cardiovascular events were found in 13.4% of the patients (4 myocardial infarctions, 4 angina pectoris, 2 strokes and 2 peripheral vascular diseases). No such events were reported in the control group (p = 0.002).

**Table 1:** Frequency of risk factors for cardiovascular disease in patients with lupus erythematosus and controls

Risk Factors	Systemic lupus	Controls	p
Mean age (years)*	34.0 (± 11.76)	35.7 (± 10.9)	0.382
Hypertension (%)*†	37.8	14.5	0.002
Diabetes mellitus (%)‡	10.9	4.8	0.233
Obesity (%)†	19.5	16.1	0.601
Over 55 years old (%)‡	2.4	4.8	0.652
Tobacco use (%)†	12.1	19.3	0.237
Family coronary disease (%)*‡	9.7	30.6	0.001
Level of high-density lipoprotein < 40 mg/dl (%)†	18.2	25.8	0.277
Level of low-density lipoprotein > 160 mg/dl (%)†	20.7	25.8	0.653
Previous ischemic events (%)*‡	13.4	0.0	0.002*

* *Two-sample Student's t test;*

† *Chi-squared test;*

‡ *Fisher's two-tailed exact test.*

*Hypertension = blood pressure > 140 × 90; obesity = body mass index > 30 kg/m^2^; family coronary disease = history of premature coronary disease in a first-degree relative < 65 years old for women and 55years old for men; previous ischemic events = stroke, myocardial infarction, angina pectoris, myocardial bypass or angioplasty, peripheral arteriopathy or aorta aneurysm.*

Focal atherosclerotic plaque was found in 41 patients (50%) and in 18 controls (29%) (p = 0.011). The comparison between patients with and without carotid plaque showed that the former were older, presented longer disease duration, higher Systemic Lupus International Collaborating Clinics scores, higher levels of LDL-c, higher frequency of diabetes, obesity, premature ovarian failure and previous cardiovascular events. No significant difference was found concerning anticardiolipin antibodies, high-density lipoprotein levels, hypertension, smoking status, family history of premature coronary disease, cumulative prednisone dose and duration of prednisone or antimalarial drug use, between patients with and without carotid plaque (Table 2).

**Table 2:** Comparison between patients with lupus erythematosus with and without carotid plaque

Variable	Plaque (n = 41)	No plaque (n = 41)	p
Age (years)*	39.9 (± 11.4)	28.2 (± 8.8)	<0.001*
Race (white %)†	53.6	46.3	0.507
Disease duration (months)*	100.2 (± 69.3)	59.8 (± 58.5)	0.005*
Hypertension (%)†	46.3	29.2	0.110
Diabetes (%)‡	19.5	2.4	0.029*
Tobacco use, (%)‡	9.7	14.6	0.737
Obesity (%)‡	36.5	2.4	<0.001*
Premature ovarian failure (%)‡	19.5	2.4	0.013*
Level of high-density lipoprotein < 40 mg/dl (%)‡	31.7	17.0	0.122
Level of low-density lipoprotein > 160mg/dl (%)‡	29.2	12.1	0.056*
Family coronary disease (%)‡	14.6	4.8	0.264
Previous ischemic events (%)‡	21.9	7.3	0.048*
Mean SLICC score*	2.1 (± 1.62)	1.0(±1.24)	<0.001*
Cumulative prednisone dose (mg)*	43,124.5 (± 32,818.2)	33,329.0 (± 31,820.2)	0.173
Prednisone use (months)*	86.0 (± 63.5)	60.5 (± 60.0)	0.065
Chloroquine use (months)*	21.9 (± 25.6)	21.7 (±33.2)	0.970

*SLICC= Systemic Lupus International Collaborating Clinics.*

* *Two-sample Student's † test;*

† *Chi-squared test;*

‡ *Fisher's two-tailed exact test;*

*Hypertension = blood pressure > 140* × *90; obesiy = body mass index > 30 kg/m^2^; family coronary disease = history of premature coronary disease in a first-degree relative < 65 years old for women and 55 years old for men; Premature ovarian failure = ovarian failure at age < 45 years; previous ischemic events = stroke, myocardial infarction, angina pectoris, myocardial bypass or angioplasty, peripheral arteriopathy or aorta aneurysm.*

In the control group, the subgroup with carotid plaque was older and there were higher percentages of systemic hypertension, obesity and tobacco use than in the subgroup without carotid plaque (Table 3). The comparison between patients and controls, both with carotid plaque, showed that the controls were older and there were higher numbers of smokers, while the patients had a higher frequency of LDL-c of over 160 mg/dl and were the only ones with previous cardiovascular events (Table 4).

**Table 3:** Comparison between controls with and without carotid plaque in relation to risk factors for cardiovascular disease and menopausal status

Variable	Plaque (n = 18)	No plaque (n = 44)	p
Age (years)*	45.6 (± 8.7)	31.7 (±8.9)	< 0.001*
Race (white %)†	50.0	59.0	0.512
Hypertension (%)‡	43.7	4.5	0.002*
Diabetes (%)‡	16.6	0.0	0.022*
Tobacco use, (%)†	38.8	11.3	0.001*
Obesity (%)‡	33.3	9.0	0.051*
Premature ovarian failure (%)‡	5.5	6.8	0.854
Level of high-density lipoprotein < 40 mg/dl (%)†	55.5	40.9	0.292
Level of low-density lipoprotein > 160 mg/dl (%)†	5.5	22.7	0.108
Family coronary disease (%)‡	16.6	36.3	0.224

* *Two-sample Student's t test;*

† *Chi-squared test;*

‡ *Fisher's two-tailed exact test.*

*Hypertension = blood pressure > 140 × 90; obesiy = body mass index > 30 kg/m2; family coronary disease = history of premature coronary disease in a frst-degree relative < 65 years old for women and 55 years old for men; premature ovarian failure = ovarian failure at age < 45 years; previous ischemic events = stroke, myocardial infarction, angina pectoris, myocardial bypass or angioplasty, peripheral arteriopathy or aorta aneurysm.*

**Table 4:** Comparison between patients with systemic lupus erythematosus and controls, both with carotid plaque

Variable	Systemic lupus (n = 41)	Controls (n = 18)	p
Age (years)*	39.9 (± 11.4)	45.6 (± 8.7)	0.043*
Race (white %)†	53.6	50.0	0.795
Hypertension (%)†	46.3	43.7	0.595
Diabetes (%)‡	19.5	16.6	0.796
Tobacco use (%)‡	9.7	38.8	0.025*
Obesity (%)†	36.5	33.3	0.810
Premature ovarian failure (%)‡	19.5	5.5	0.169
Level of high-density lipoprotein < 40 mg;dl (%)†	31.7	55.5	0.083
Level of low-density lipoprotein > 160 mg/dl (%)†	29.2	5.55	0.043
Family coronary disease (%)‡	14.6	16.6	0.842
Previous ischemic events (%)‡	21.9	0.0	0.001*

* *Two-sample Student's t test;*

† *Chi-squared test;*

‡ *Fisher's two-tailed exact test.*

*Hypertension = blood pressure > 140 × 90; obesity = body mass index > 30 kg/m^2^; family coronary disease = history of premature coronary disease in a first-degree relative < 65 years old for women and 55 years old for men; premature ovarian failure = ovarian failure at age < 45 years; previous ischemic events = stroke, myocardial infarction, angina pectoris, myocardial bypass or angioplasty, peripheral arteriopathy or aorta aneurysm.*

The risk factors associated with carotid plaque in patients and controls, using logistic regression, were: older age (odds ratio, OR: 1.12; 95% confidence interval, CI: 1.07- 1.17), obesity (OR: 6.16; 95% CI: 1.84 — 20.64) and the diagnosis of systemic lupus erythematosus (OR: 4.74; 95% CI: 1.83 — 12.27) (Table 5). In the logistic regression model that included only patients and lupus-related risk factors, higher Systemic Lupus International Collaborating Clinics scores (OR: 1.69; 95% CI: 1.13 — 2.52) and longer disease duration (OR: 1.65; 95% CI: 1.06 — 2.56) were independently associated with carotid plaque (Table 6).

**Table 5:** Variables associated with carotid plaque among 144 participants who underwent B-mode ultrasound examination, 82 with lupus erythematosus and 62 controls

Explanatory variables	Beta coefficient	Odds ratio	95% confidence interval	p
Age (years)*	0.11	1.12	1.07-1.17	< 0.001
Obesity	1.81	6.16	1.84-20.64	0.003
Diagnosis of systemic lupus	1.55	4.74	1.83-12.27	0.001

* *Estimates based on one-year age increments. Obesity = body mass index > 30 kg/m^2^.*

**Table 6:** Lupus-related risk factors for coronary disease in 82 patients

Explanatory variables	Beta coefficient	Odds ratio	95% confidence interval	p
Systemic Lupus International Collaborating Clinics score*	0.52	1.69	1.13-2.52	0.010
Duration of lupus†	0.50	1.65	1.06-2.56	0.025

* *Estimates based on one-point increments in the Systemic Lupus International Collaborating Clinics score;*

† *Estimates based on one-year increments in disease duration.*

## DISCUSSION

This is the first study evaluating the prevalence of subclinical carotid atherosclerosis using B-mode ultrasound among patients with systemic lupus erythematosus from a population with different ethnic mixes in a developing country. Our patients were younger than patients evaluated in other studies using the same method^12-16^ but, nevertheless, we found a higher prevalence of atherosclerotic plaque. The logistic regression analysis showed that lupus *per se* was an independent risk factor for carotid plaque. This finding has also been reported by another group.^15^

The patients and controls evaluated in this study were very similar with regard to age, race and mean number of risk factors for coronary disease. However, there were more patients with three or more risk factors for coronary disease and they were the only ones with previous cardiovascular events. Arterial events were observed in twelve patients (13.4%), which is comparable to the prevalence of 15% reported by Manzi et al.^12^ in another cross-sectional study and slightly higher than the prevalence of cardiovascular events observed in some other lupus cohorts, in which the range was from 7.4 to 8.8%.^2-4^

Although the patients in our study were younger than in other studies that have evaluated the prevalence of carotid atherosclerosis, we found a 50% prevalence of carotid plaque. In other studies on lupus patients this prevalence ranged from 32 to 41%.^12-16^ These differences may be related to differences in lifestyle and genetic background, between our patients and those in other studies. This is a still open question that needs to be further addressed.

The prevalence of carotid plaque in our controls was higher than what was reported by Roman et al. (9%)^13^ and Svenungsson et al. (11.5%).^14^ However, their samples were smaller. Our result is similar to the one reported by the Women's Healthy Lifestyle Project at Pittsburgh University, which found 25.5% carotid plaque in 208 women,^12^ and is also similar to results from other studies among general populations.^22-24^

The prevalence of carotid plaque has been higher (50 to 67%) in studies that included middle-aged and post-menopausal women, and this probably reflects the influence of age.^23,24^ However, with regard to the influence of menopausal status, the present study is the first to demonstrate that premature ovarian failure is more frequently found among patients with systemic lupus erythematosus who present carotid plaque.

The cause of higher prevalence of carotid atherosclerosis in lupus patients may be attributed to a cluster of risk factors. However, in the present study there was not much difference in the mean number of risk factors between patients and controls.

Rahman et al., comparing 35 patients with systemic lupus erythematosus and 397 controls with angiographically proven premature coronary disease found a lower mean number of risk factors among patients with lupus than among controls.^25^ Esdaile et al., evaluating risk factors for coronary disease among participants in two Canadian lupus cohorts by means of the Framingham multiple logistic regression model, still found a high risk of developing coronary disease among patients with systemic lupus, after removing the influence of traditional risk factors for coronary disease.^26^ The evidence found by Rahman et al. and Esdaile et al. and the results from our study suggest that there might be an influence on systemic lupus erythematosus from risk factors for atherosclerotic events other than the traditional risk factors for coronary disease.

In the present study, the traditional risk factors independently associated with carotid plaque were older age at the time of the study and obesity. Roman et al. found no difference related to traditional risk factors among lupus patients with and without carotid plaque.^13^ Other studies have shown that there is an influence on ischemic heart events in systemic lupus from the traditional risk factors for coronary artery disease. However, these studies had relatively small samples, in comparison with studies among general populations, and they used different definitions for certain risk factors.^5^ Lupus cohorts have especially shown the influence of hypercholesterolemia and hypertension on clinical coronary disease.^2-4^ In Manzi's study, the risk factors independently associated with carotid plaque in systemic lupus erythematosus were older age, raised systolic blood pressure, higher levels of low-density lipoprotein and a previous coronary event.^12^ Svenungsson found lower levels of high-density lipoprotein, raised plasma concentrations of oxidized low-density lipoprotein, raised triglycerides, lipoprotein (a), homocysteine and lupus anticoagulant, more osteoporosis and higher cumulative prednisone dose among patients with previous coronary disease, in comparison with patients without coronary disease.^14^

In a study that included 197 lupus patients, Roman et al. found that older age and higher levels of total cholesterol were independent risk factors for carotid plaque.^15^ In another recent study, Selzer et al. found that the determinants of carotid plaque were older age, higher systolic blood pressure, higher levels of high-density lipoprotein and antidepressant use.^16^

In the present study, carotid plaque in the control group seemed to be more influenced by traditional coronary disease risk factors than in patients with lupus. Older age, diabetes, hypertension, obesity and current smoking were more common among controls with carotid plaque. The comparison between patients and controls, both with carotid plaque, showed that the patients were younger than the controls.

Among the systemic lupus erythemato-sus-related variables analyzed in this study, longer disease duration and higher Systemic Lupus International Collaborating Clinics score were associated with carotid plaque. In the latter, we also included cardiovascular disease and therefore it may be biased. We could not find associations for the presence of carotid plaque, with duration of prednisone use, cumulative prednisone dose, duration of chloroquine use or the presence of anticardiolipin antibodies. However, Manzi et al. reported an association between longer duration of prednisone use and carotid plaque^12^ and Svenungsson et al. found higher cumulative prednisone dose in lupus cases.^14^ In different lupus cohorts, older age at diagnosis, longer disease duration and longer duration of steroid therapy have been associated with coronary disease.^2-4^

## CONCLUSION

In this study, the higher prevalence of carotid plaque among patients with systemic lupus erythematosus who were even younger than those previously studied has confirmed that there is also a pattern of accelerated atherosclerosis among women with lupus in an ethnically mixed population from a developing country. The data from different studies, relating to possible risk factors involved in this higher prevalence of atherosclerosis in lupus patients, are still conflicting. We found that the prevalence of carotid plaque was significantly associated with older age, obesity, longer disease duration and higher Systemic Lupus International Collaborating Clinics scores. Furthermore, a diagnosis of systemic lupus was demonstrated to be an important risk factor for the presence of carotid atherosclerosis. Although it is difficult to modify many of the risk factors, such as diet, efforts need to be made towards reducing the risk factors for coronary disease among patients with systemic lupus erythematosus, so as to prevent atherosclerotic events.
